# Mothers’ willingness to pay for HPV vaccines in Anambra state, Nigeria: a cross sectional contingent valuation study

**DOI:** 10.1186/s12962-016-0057-0

**Published:** 2016-06-06

**Authors:** Ifeoma Blessing Umeh, Sunday Odunke Nduka, Obinna Ikechukwu Ekwunife

**Affiliations:** Department of Clinical Pharmacy and Pharmacy Management, Nnamdi Azikiwe University, Awka, Nigeria; Collaborative Research Group for Evidence-Based Public Health, Department of Prevention and Evaluation, Leibniz Institute for Prevention Research and Epidemiology-BIPS/University of Bremen, Achterstr. 30, 28359 Bremen, Germany

**Keywords:** Uterine cervical neoplasms, Papilloma virus vaccine, Willingness-to-pay, Patient preference, Nigeria

## Abstract

**Background:**

Human papilloma virus (HPV) vaccination in Nigeria will require substantial financing due to high cost of HPV vaccine and inexistence of structures to support adolescent vaccination. Alternative sources are needed to sustain the government funded HPV vaccination programme. This study assessed Nigerian mothers’ willingness-to-pay (WTP) for HPV vaccine. We also compared the difference between the average WTP and estimated costs of vaccinating a pre-adolescent girl (CVG).

**Methods:**

We conducted a quantitative, cross-sectional, survey-based study in which 50 questionnaires were distributed to each of 10 secondary schools located in two rural and one urban city in Anambra state. The questionnaires were then randomly distributed to girls aged 9–12 years of age to give to their mothers. Contingent valuation approach using the payment card technique was used to estimate the average maximum WTP among the survey participants. Correlates of WTP for HPV vaccination were obtained using multivariate logistic regression. Estimated CVG was obtained by adapting cost of HPV vaccine delivery in Tanzania to the Nigerian setting.

**Results:**

A total of 438 questionnaires (88 %) were returned. The average WTP was US$ 11.68. This is opposed to estimated delivery cost of US$ 18.16 and US$ 19.26 for urban and rural populations respectively at vaccine price offered by the Vaccine Alliance (Gavi) and US$ 35.16 and US$ 36.26 for urban and rural populations respectively at the lowest obtainable public sector vaccine price. Demand for HPV vaccine was deemed high (91.6 %) and was significantly associated with respondents previously diagnosed of HPV infection.

**Conclusion:**

Demand for HPV vaccine was high although short of estimated CVG. High demand for vaccine should be capitalized upon to increase vaccine uptake. Education on cervical cancer and provider-initiated vaccination should be promoted to increase vaccine uptake. Co-payment could be a feasible financing strategy in the event of national HPV vaccination.

## Background

Cervical cancer is the fourth most commonly diagnosed cancer and fourth leading cause of cancer related deaths among females worldwide [1]. Every year, about 528,000 women around the world are diagnosed with cervical cancer and about 266,000 women die from the disease [1]. Cervical cancer ranks as the second most frequent cancer among women in Nigeria, with about 47 million women aged 15 years and older being reported to be at risk of developing cervical cancer. Further, almost 14,089 Nigerian women are diagnosed with cervical cancer and 8240 die from the disease yearly [2]. The health and economic burden of cervical cancer is hence substantial. Cervical cancer deaths often occur in relatively young women, who are raising children, caring for families, and contributing to communities [3].

While cervical cancer screening programmes have been effective in reducing cervical cancer incidence in developed countries, cervical cancer screening in Nigeria is still unpopular [2]. There is currently no organized national screening programme in the country. HPV screening is largely opportunistic, with screening coverage estimated to be around 8.7 % [2]. The two types of vaccines that prevent cervical cancer—GSK’s bivalent HPV vaccine (Cervarix) and Merck & Co. Inc.’s quadrivalent HPV vaccine (Gardasil)—are both licensed in Nigeria. The vaccines are highly effective in preventing persistent HPV infection and subsequent precancerous lesions due to infection with two types of HPV—types 16 and 18—that cause about 70 % of cervical cancer worldwide [2].

High prices have been a major barrier to introducing HPV vaccines in developing countries. The Vaccine Alliance (Gavi) and its partners provide the poorest countries with access to a sustainable supply of new and underused vaccines. Gavi offers access to HPV vaccines for as little as US$ 4.50 per dose and provides support for HPV demonstration programmes and the national introduction of HPV vaccines [4]. The type of support provided however depends on a country’s demonstrated ability to deliver vaccines to young adolescent girls [4]. Gavi’s current vaccine support for Nigeria includes pentavalent, pneumococcal conjugate, yellow fever, meningitis A and measles vaccines, as well as cash support for health system strengthening and immunization system strengthening [5].

Currently, HPV vaccines are purchased ‘out-of-pocket’ and are not included among the vaccines offered for free under the National Immunization Programme (NIP) in Nigeria. Providing free HPV vaccination would further strain the government’s tight health budget. Beside the cost of the vaccine, HPV vaccination requires the development of a new vaccine delivery service for adolescent girls in order to achieve the required doses [6]. This is particularly due to the lack of an existing structure to support the adolescent vaccine delivery. Therefore, even with Gavi’s support, HPV vaccination will require a substantial sum of money for its delivery.

The National Primary Health Care Development Agency (NPHCDA) in Nigeria recently announced the commencement of plans for national HPV vaccination. The plan involves a demonstration project in some selected states of the federation in order to determine cost, acceptance and best delivery method [7]. A financially sustainable HPV vaccine delivery strategy is likely to require user financing to offset some percentage of the actual cost of vaccination. However, the viability of user fees as a financing mechanism for HPV vaccine depends on private demand for the vaccines. Several studies that assessed parental acceptance of HPV vaccination for their daughters in Nigeria reported high levels of acceptance for the vaccine [8–10]. However, to the best of our knowledge, no study has estimated the amount parents are willing to pay for the HPV vaccine. The objective of this study was to assess Nigerian mothers’ willingness-to-pay (WTP) for the HPV vaccine using a contingent valuation method. We also compared the difference between the average WTP value and estimated cost of vaccinating a pre-adolescent girl (CVG) against HPV infection in Nigeria. We focused on mothers because the responsibility of family health in many families mainly falls to the mother [11].

## Methods

### Study design and study population

This study, a school-based cross sectional survey was conducted in Anambra state, Nigeria, from February to August, 2015. Anambra state was selected due to its accessibility and proximity to the researchers. The state is located at latitude 6.20°N and 7.00°E with total area of 4844 km and population of 4.1 million [12]. The main indigenous ethnic group in Anambra state is Ibo and there is also a small population of Igala. The state contains numerous thickly populated villages and small towns. Anambra state is rich in natural gas, crude oil, and bauxite and ceramic and has almost 100 % arable soil. Literacy level in the state is quite high. The inhabitants are mainly business people, government workers and students. According to the State Ministry of Education record, there are 254 public secondary schools and 166 private secondary schools in the state.

Ten secondary schools (five schools in Onitsha, three schools in Ekwulobia and two secondary schools in Isuofia) all in Anambra state were purposively selected for the study. Onitsha is an urban city while Ekwulobia and Isuofia are rural cities. Five schools were privately owned while the other five were public schools. The reason for selecting schools from both urban and rural area as well as from both private and public schools was to ensure inclusion of persons from all socioeconomic strata. Eligibility for participation (i.e. to be given a questionnaire) was (1) female students aged between 9 and 12 years old and (2) their mothers being able to read and write in English language.

Using Anambra state population size of 4.1 million [12], confidence level of 95 % and margin of error of 3 %, 385 respondents (approximately 40 respondents/school) were determined to be appropriate for the survey. Fifty questionnaires were distributed to each of the 10 secondary schools to be distributed randomly to eligible girls.

The questionnaires were given to school teachers who distributed them to girls aged 9–12 years to take home to their mothers. The mothers were requested to return the completed questionnaire via their child back to the school teachers within 3–7 days. Contingent valuation approach using the payment card technique was used to estimate the average maximum WTP among the survey participants.

### Willingness-to-pay for HPV vaccine assessment

A 23-item self administered questionnaire was developed for the WTP assessment. The questionnaire consisted of three sections. The first section included general information and socio-demographic characteristics such as age, number of daughters, level of education etc. The second section assessed awareness of HPV, genital warts, cervical cancer as well as HPV vaccines. Five questions examining causes of genital warts and cervical cancer were used to assess the knowledge of those aware of the diseases and HPV vaccine (i.e. knowledge index score). The third section presented facts about HPV and contained the payment card used to assess mothers’ WTP for HPV vaccine.

Vaccine rejection was measured based on the response to the following question: “If the vaccine is not free, and you have to pay ‘out of pocket’ by yourself, will you vaccinate your daughter against HPV”? The follow-up question was used to assess willingness to pay (WTP) of “vaccine acceptors”. The question reads as follows: “If so, from the scale below mark ‘*x’* on the maximum amount you will pay (in Naira) to have your daughter vaccinated against HPV”. The parents who answered “no” or indicated zero in the payment card were classified as “vaccine rejecters”, while the ones who answered “yes” and indicated a positive value in the payment card were classified as “vaccine acceptors”. Offered WTP values in the payment card ranged from zero to more than 12,000 Naira (equivalent to US$ 60). The maximum price offered reflects the Nigerian market price for the vaccine. The maximum amount they were willing to pay was considered as their perceived monetary benefit of the vaccine. This is in accordance with welfare economic theory which states that the benefit to an individual of a service or intervention is defined as the individual’s maximum willingness to pay for the service or intervention.

### Ethical consideration

The research design and procedure were approved by the ethical clearance committee of Nnamdi Azikiwe University Teaching Hospital Nnewi, Anambra state. The study secured written informed consent from the respondents. Anonymity of participants’ data was maintained by not including participants’ names.

### Data analysis

Responses to the willingness-to-pay (WTP) question were grouped into two categories: ‘vaccine acceptors’ versus ‘vaccine rejecters’. The response to WTP question served as the dependent variables in multivariate binary logistic regression. The explanatory or independent variables were re-categorized into the following variables:

1. Socio-economic data: place of residence (urban or rural); age of respondents (three dummy codes for 31–40 years, 41–50 years, and >50 years); household size (three dummy codes for 4–6 persons, 7–9 persons and ≥10 persons); occupation; average household income (4 dummy codes for US$ 251–502 versus others, US$ 503–1256, US$ 1257–2512, and >US$ 2513); whether respondent is religious; whether respondent is a catholic; and whether respondent is a protestant.
2. Awareness of HPV infection: ever diagnosed of infection, ever diagnosed of genital warts, and knowledge of HPV infection and consequences (summarized by differentiating those that answered all questions on knowledge of HPV infection correctly from those that did not).

Data were initially coded and transferred to Microsoft Excel (Microsoft Office 2010). Further re-categorization of data (i.e. creation of dummy variables) was done in Microsoft Excel before importing the data to SPSS (Version 20). Multivariate binary logistic regression used the backward conditional as enter method and was performed with SPSS version 20. A two-tailed significance value of 0.05 was used.

#### Estimation of average cost per vaccinated girl

Cost per vaccinated girl (CVG) was estimated by adjusting cost of HPV vaccination delivery in Tanzania to the Nigerian setting [13]. We adjusted the Tanzanian HPV delivery cost estimates by modifying cost items—social mobilization/information, training, procurement (except for vaccine cost), vaccination, cold storage, and administration/supervision—based on the difference in local purchasing power between Tanzanian and Nigeria. Difference in local purchasing power was computed with a web-based cost of living calculator [14]. In accordance with recent recommendations, vaccine procurement cost was modified to reflect the cost of two doses at $4.50 per dose instead of 3 doses at $5 as per original study [15]. Vaccine price of US$ 4.50 reflects the price being offered by Gavi for countries eligible for support, while vaccine price of U$ 13 represents the lowest public sector price offered by HPV vaccine manufacturers [4]. Except for the vaccine cost, all other costs were inflated from 2012 (i.e. the year of publication) to 2015 US$ value. This was done by converting adjusted cost in US$ to naira equivalent, inflating to 2015 value using the consumer price index, and then converting back to US$. We used exchange rates published by Central bank of Nigeria [16] and consumer price index published by World Bank [17].

## Results

### Characteristics of respondents and their awareness of HPV infection

The overall response rate was 88 %. More than half of the respondent (57.1 %) resided in the rural areas. The majority of the mothers who participated in the survey were between the ages of 31 and 50 years and were mainly from the Ibo tribe. Thirty percent of the respondents completed primary school, 38.2 % completed secondary school, 17.2 % had tertiary education while 10.3 % had post tertiary education. Only 4.3 % of respondents had no formal education. More than half of the respondents (57.6 %) reported having a household monthly income of less than <US$ 251. All the respondents except one person had a form of religious belief. One half of the respondents were Catholics (Table 1).

**Table 1:** Socioeconomic characteristics of respondents (n = 438)

Variable	Frequence (%) or average (±SD)
*Place of residence*	
Rural	250 (57.1)
Urban	188 (42.9)
*Age of mothers (years)*	
<30	65 (14.9)
31–40	169 (38.8)
41–50	157 (36.0)
>50	45 (10.3)
*Number of daughters*	
One	314 (71.9)
Two	106 (24.3)
Three	12 (2.7)
Four	5 (1.1)
Average age of daughters	10.7 (±1.4)
*Tribe of respondent*	
Ibo	422 (96.8)
Others	14 (3.2)
*Level of education*	
No education	19 (4.3)
Primary education	131 (30.0)
Secondary education	167 (38.2)
Tertiary education	75 (17.2)
Post tertirary education	45 (10.3)
Monthly income^a^	
Less than <US $251 (N50,000)	251 (57.6)
US$ 251–502 (N50,000–N100,000)	109 (25.0)
US$ 502–1256 (N100,000–N250,000)	43 (9.9)
US$ 1256–2512 (N250,000–N500,000)	16 (3.7)
>US$ 2512 (>N500,000)	17 (3.9)
*Occupation*	
No occupation	28 (6.4)
Farming	57 (13.1)
House wife	40 (9.2)
Public servant	110 (25.3)
Private business	197 (45.3)
Others	2 (0.5)
*Religious preference*	
None	1 (0.2)
Traditionalist	48 (11.0)
Catholic	276 (63.4)
Protestant	101 (23.2)
Muslim	7 (1.6)
Others	2 (0.5)

^a^1 US$ = 199 Nigerian Naira

Table 2 shows details of awareness of HPV infections and its consequences. Very few respondents had had or been diagnosed with either HPV infection or genital warts. Also, very few mothers had heard of HPV infection (19.1 %). Among the population that were aware of HPV infection, only very few had good knowledge of HPV infection and its consequences according to our knowledge index score.

**Table 2:** Awareness of HPV infection and consequences (n = 438)

Variable	Frequency (%)
Diagnosed with HPV infection	33 (7.6)
Diagnosed with genital warts	31 (7.1)
Ever heard of HPV infection	83 (19.1)
*Sources of HPV infection awareness*	
Doctor, nurse or health professional	27 (32.9)
Family or friends	11 (13.4)
Newspaper or magazine	12 (14.6)
Television	11 (13.4)
Internet	7 (8.5)
Cannot remember	7 (8.5)
Multiple sources	7 (8.5)
Ever heard of cervical cancer	122 (27.9 %)
Good awareness of HPV infection and consequences	15 (3.4 %)

### Willingness to pay (WTP) for HPV infection

#### Average WTP value

As shown in Table 3, 401 of the respondents (91.6 %) stated a positive WTP amount. The average WTP amount stated by the respondents was US$ 5.84 per dose while the most frequently stated amount was US$ 2.51 per dose. Fifty percent of the respondents stated US$ 5.03 as the amount they are willing to pay for a dose of HPV vaccine.

**Table 3:** WTP amount for HPV vaccines and its predictors

Statistics	WTP per dose (US$)
Mean	5.84
Median	5.03
Mode	2.51
*Percentiles*	
20	2.51
90	7.54

	Dependent: vaccine acceptance (=1)		
	b	S.E	b (exp)
*Vaccine rejection, n* *=* *33*			
Residence (urban)	−1.30	0.82	0.27
Diagnosed with HPV infection	18.76	8358.68	100,000,000
Constant	−2.83	1.04	21.50
Nagelkerke R^2^	0.13		

#### Logistic regression predicting willingness-to-pay for vaccine

Thirty-three of the respondents (7.5 %) rejected HPV vaccination of their daughters (Table 3). Logistic regression showed that mothers living in an urban area were 1.3 times less likely to demand for HPV vaccination for their daughters. Also mothers that have been previously diagnosed of HPV infection were 18.8 times more likely to demand for HPV vaccination for their daughters. The predictive capacity of the model was 13 %.

#### Estimated average cost per vaccinated girl

Estimate of CVG was adapted from an HPV vaccine delivery pilot project in Tanzania and showed that if HPV vaccine is supplied at vaccine alliance’s (Gavi) price, cost per vaccinated girl (CVG) in urban and rural area could cost as much as US$ 18.16 and US$ 19.26 respectively. However, CVG could be as high as US$ 35.16 and US$ 36.26 for urban and rural areas respectively, if vaccine is supplied at the lowest price which the vaccine manufacturers have offered the vaccine to the public sector. Details of estimated CVG are shown in Table 4.

**Table 4:** Estimated economic costs per fully-immunized girl (US$) in a scaled-up regional school based HPV vaccination programme

Cost items	Source data (Mwanza vaccine project, Tanzania) [13]		Gavi vaccine price (US$ 4.5) [4]		Lowest public sector price (US$ 13) [4]	
	Urban	Rural	Urban	Rural	Urban	Rural
Social mobilization/IEC	0.5	0.5	0.44	0.44	0.44	0.44
Training	0.3	0.5	0.26	0.44	0.26	0.44
Procurement^a^	18.7	19.4	12.46	13.11	29.46	30.11
Vaccination	5.0	4.4	4.39	3.87	4.39	3.87
Cold storage	0.2	0.3	0.18	0.26	0.18	0.26
Waste management	0.0	0.0	0.0	0.0	0.0	0.0
Admin/supervision	0.5	1.3	0.44	1.14	0.44	1.14
Total	25.3	26.6	18.16	19.26	35.16	36.26

^a^Vaccine procurement cost was modified to reflect the cost of two doses at US$4.5 per dose instead of three doses at US$5 as per original study

## Discussion

This study aimed to establish how much Nigerian mothers are willing to pay for vaccination of their daughters against HPV infection. The findings showed that the majority of the mothers were willing to pay an average of US$ 11.68 to get their daughters fully vaccinated. Mothers that were previously diagnosed of HPV infection were more likely to demand for the vaccine. Mothers that live in urban areas were less likely to demand for the vaccine. The shortfall needed to augment the cost of vaccination ranges from US$ 6.48 to US$ 24.58 depending on the setting of vaccine delivery (rural or urban) and the unit cost of HPV vaccine (Gavi’s price or lowest price offered to public sector).

This study has useful implications for increasing uptake of HPV vaccine and planning HPV vaccination in Nigeria. Firstly, demand for HPV vaccine is quite high. A total of 91.6 % of mothers were willing to pay for HPV vaccination of their daughters. This is opposed to the fears of HPV vaccine rejection that is speculated on due to cultural and religious sensitivities towards health interventions that target prevention of a sexually transmitted disease [18]. For instance, it has been stated that mothers may be concerned about the vaccine being a ‘license to premarital sex’ [18]. Other Nigerian based population studies have also reported high HPV vaccine acceptance. A study conducted among female health care workers in Enugu, South-Eastern Nigeria reported HPV vaccine acceptability rate of 91.0 % [8]. HPV vaccine acceptance of 74 % among female university students in northern Nigeria has been reported [19]. Also, 70 % accepted vaccination of their daughters in a cross-sectional survey of mothers attending the gynaecology clinic in a Nigeria University Teaching Hospital [10]. High acceptance and demand for the vaccine points to high likelihood of HPV vaccination programme success in Nigeria as fundamental to the success of such programme is the recipients’ willingness to accept the vaccine.

The high demand for HPV vaccines could be capitalized upon to increase utilization of the vaccines in Nigeria. It could be possible to achieve higher vaccine uptake in Nigeria without relying on the government to provide the vaccine. A possible solution to achieving higher uptake of the vaccine is through education of the populace about cervical cancer and HPV vaccination. For example, those diagnosed of HPV infection were more likely to demand for HPV vaccination of their daughters. This group of persons will most likely have more in-depth knowledge of the disease and thus, explains their disposition towards HPV vaccine. A systematic review by Kessels et al. [20] also identified having higher vaccine-related knowledge, having a healthcare provider as a source of information and maintaining positive vaccine attitudes as correlates of HPV vaccine uptake in teenage girls. While waiting for national immunization, interventions that improve understanding of, and positive attitudes toward HPV vaccine could be applied. Educational interventions directed to parents or to adolescents/young adults have been shown to be moderately effective in increasing HPV vaccine acceptance and uptake [21]. Simple educational interventions such as fact sheet about epidemiology and morbidity associated with HPV infection or a few minutes of radio novel about cervical cancer case will help increase vaccine uptake in Nigeria [21].

Another solution to achieving high HPV vaccine uptake is to properly orient health professionals to inform patients and the public about HPV vaccination. Even if HPV vaccination is free under the national immunization programme, uptake of the vaccine will basically depend on whether health professionals are willing to inform adolescent girls about HPV vaccination. Provider-initiated model for improving health service utilization appears to be effective in developing countries. For example, increase in HIV test rates have been attributed to the rapid scale-up of the provider-initiated testing model [22]. The prescriber’s ability to educate their patient population on HPV vaccination must be supported and included as part of the national strategic plan for cervical cancer control. Denmark achieved very high vaccination rates (3-dose coverage of over 80 %) through administration by general practitioners [23].

The second important finding from our study is that co-payment for HPV vaccination could be a viable option to augment the cost of vaccination in a government funded vaccination scenario. HPV vaccination is a costly programme. Even at Gavi vaccine prices, HPV vaccination requires substantial set up costs. Unlike new infant vaccines, which may be added to an existing infant vaccine delivery system, HPV vaccination requires the development of a new vaccine delivery service in order to achieve the required doses [6]. This is particularly so because of: (1) micro-planning defined as planning of vaccination activities at local levels that take into account issues of accessibility, geography, population movements, and cultural characteristics; (2) social mobilization/information, education and communication; (3) higher cold chain equipment requirements for delivery outside health facility; and (4) higher service delivery costs [6]. With health expenditure of US$ 115 per capita and considering other competing health services including nutrition activities and emergency aid [24], it is important to consider other financing options to support HPV vaccination programme in Nigeria.

The caveat to co-payment as an option of financing HPV vaccination is that it may skew the vaccination programme to favour only those that can afford the vaccine. US$ 11.68 could be catastrophic for many to afford in Nigeria considering that about 99 million Nigerians or about 58 % of the population live with less than US$ 1.25 per day [25]. In order to ensure equity balanced programme, HPV vaccination should be provided for free to the poor populace in Nigeria. As a suggestion, a practical way to achieve this is to stratify different secondary schools based on their school fees and offer free vaccination to schools in lowest stratum in school-based vaccine delivery system. Poor populations e.g. rural dwellers could be targeted for free vaccination in outreach vaccine delivery systems.

The WTP value obtained in the study has to be considered in the light of bias that is associated with open-ended elicitation formats and WTP surveys in general. Respondents could have been influenced by the range of values chosen for the payment scale question design rather than their true maximum WTP values. It is also possible that some respondents may have stated no WTP value or very low WTP value especially if they feel that vaccination should be paid by the government. The small sample size of vaccine rejecters could have induced a systematic bias as logistic regression could overestimate odd ratios in studies with small to moderate sample size [26]. Despite these limitations, this is the first study that assessed mother’s WTP for HPV vaccination in Nigeria. The timeliness of this study makes the findings useful for HPV vaccination planning in Nigeria.

## Conclusion

The findings showed that Nigerian mothers were willing to pay an average of US$ 11.68 for HPV vaccination of their daughters. At Gavi vaccine price, US$ 6.48 and US$ 7.58 extra are needed to augment the cost of vaccination in urban and rural areas respectively. At the lowest obtainable public sector vaccine price, US$ 23.48 and US$ 24.58 extra are needed to augment the cost of vaccination in urban and rural areas respectively. Demand for HPV vaccine was high and this should be capitalized upon to increase uptake of HPV vaccine. Educating the populace on cervical cancer and provider-initiated vaccination should be promoted as these could increase HPV vaccine uptake. In the event of government funded national vaccination, co-payment could be a feasible strategy to ensure sustenance of vaccination. However, free vaccination should be considered for the poor populace in order to ensure equity sensitive vaccination programme.
